# Loaded delta-hemolysin shapes the properties of *Staphylococcus aureus* membrane vesicles

**DOI:** 10.3389/fmicb.2023.1254367

**Published:** 2023-10-06

**Authors:** Juan Chen, Yuhuan Lv, Weilong Shang, Yi Yang, Yuting Wang, Zhen Hu, Xiaonan Huang, Rong Zhang, Jizhen Yuan, Jingbin Huang, Xiancai Rao

**Affiliations:** ^1^Department of Pharmacy, The Second Affiliated Hospital, Army Medical University, Chongqing, China; ^2^Department of Microbiology, College of Basic Medical Science, Army Medical University, Chongqing, China; ^3^Department of Clinical Laboratory, The 971st Hospital of Chinese People's Liberation Army Navy, Qingdao, China

**Keywords:** *Staphylococcus aureus*, membrane vesicle, Hld, agrA, inflammatory factor

## Abstract

**Background:**

Membrane vesicles (MVs) are nanoscale vesicular structures produced by bacteria during their growth *in vitro* and *in vivo*. Some bacterial components can be loaded in bacterial MVs, but the roles of the loaded MV molecules are unclear.

**Methods:**

MVs of *Staphylococcus aureus* RN4220 and its derivatives were prepared. Dynamic light scattering analysis was used to evaluate the size distribution, and 4D-label-free liquid chromatography–tandem mass spectrometry analysis was performed to detect protein composition in the MVs. The site-mutation *S. aureus* RN4220-Δhld and *agrA* deletion mutant RN4220-ΔagrA were generated via allelic replacement strategies. A hemolysis assay was performed with rabbit red blood cells. CCK-8 and lactate dehydrogenase release assays were used to determine the cytotoxicity of *S. aureus* MVs against RAW264.7 macrophages. The serum levels of inflammatory factors such as IL-6, IL-1β, and TNFα in mice treated with *S. aureus* MVs were detected with an enzyme-linked immunosorbent assay kit.

**Results:**

Delta-hemolysin (Hld) was identified as a major loaded factor in *S. aureus* MVs. Further study showed that Hld could promote the production of staphylococcal MVs with smaller sizes. Loaded Hld affected the diversity of loaded proteins in MVs of *S. aureus* RN4220. Hld resulted in decreased protein diversity in MVs of *S. aureus*. Site-mutation (RN4220-Δhld) and *agrA* deletion (RN4220-ΔagrA) mutants produced MVs (^Δ*hld*^MVs and ^Δ*agrA*^MVs) with a greater number of bacterial proteins than those derived from wild-type RN4220 (^wt^MVs). Moreover, Hld contributed to the hemolytic activity of ^wt^MVs. Hld-loaded ^wt^MVs were cytotoxic to macrophage RAW264.7 cells and could stimulate the production of inflammatory factor IL-6 *in vivo*.

**Conclusion:**

This study presented that Hld was a major loaded factor in *S. aureus* MVs, and the loaded Hld played vital roles in the MV-property modification.

## 1. Introduction

Bacterial membrane vesicles (MVs) are nanosized bilayer lipid particles that are commonly released from bacterial cells during all their growth stages (Wang et al., 2021). Gram-negative *Escherichia coli* was first reported in the 1960s to secrete MVs, which are considered to bud out from the outer membrane, so they are often termed outer MVs (OMVs). Conversely, the existence of MVs released from gram-positive bacteria such as *Bacillus subtilis* and *B. cereus* was first mentioned in 1990 (Brown et al., 2015; Kaparakis-Liaskos and Ferrero, 2015; Bitto et al., 2021a). Released MVs can incorporate bacterial proteins, lipids, and genetic materials (Brown et al., 2015), and these properties confer bacterial MVs with versatile function particles for surface adhesion, cell signal transduction, virulence factor transportation, genetic material exchange, and bacterial pathogenicity, as well as for applications in vaccine candidate and drug delivery (Askarian et al., 2018; Briaud and Carroll, 2020; Escudé Martinez de Castilla et al., 2021; Kumaraswamy et al., 2021; Lee et al., 2022).

*Staphylococcus aureus* is a notorious pathogen causing various diseases, and the release of MVs by *S. aureus* was first discovered in 2009 (Lee et al., 2009). The function of *S. aureus* MVs may be strain-oriented. For example, MVs derived from *S. aureus* ATCC14458 can induce atopic dermatitis-like skin inflammation (Hong et al., 2011), clinical *S. aureus* isolate 06ST1048-produced MVs can induce the apoptosis of HEp-2 cells in a dose-dependent manner, and MVs prepared from ST239, ST59, and ST338 methicillin-resistant *S. aureus* strains could inhibit the proliferation and induce the apoptosis of epithelial cells HaCaT (Gurung et al., 2011; Chen et al., 2023); MVs of *S. aureus* strain NCTC6571 and clinical isolates BPH2760 and BPH 2900 can induce autophagy in A549 lung epithelial cells (Bitto et al., 2021a). MVs produced from *S. aureus* USA300-ΔagrA can also bind and inactivate daptomycin to protect bacteria from antibiotic killing (Pader et al., 2016). A study compared the cytopathology of host cells induced by MVs derived from different *S. aureus* strains and concluded that MVs with different proteomes presented distinct cytotoxic effects against host cells (Jeon et al., 2016). Importantly, delta-hemolysin (Hld) was found in the MVs from three *S. aureus* isolates M060, 03ST17, and 06ST1048, and the loaded Hld could induce cytotoxicity after delivery to host cells by MVs (Jeon et al., 2016). However, the biological effects of components encapsulated in *S. aureus* MVs such as Hld on MV properties and functions are largely unknown.

Certain components such as toxins and virulence factors can be concentrated in bacterial MVs (Rivera et al., 2010; Veith et al., 2014). Investigating the biological roles of loaded molecules in MVs has great significance for elucidating MV properties and facilitating its applications. The coding gene of Hld is located at the 85–165 bases of *RNAIII*, an RNA regulator involved in *S. aureus agr* locus carrying *agrB, agrD, agrC, agrA*, and *RNAIII* (Hodille et al., 2016). Hld is a hemolytic peptide belonging to the phenol-soluble modulin (PSM) family that contributes to *S. aureus* infections (Verdon et al., 2009). In the present study, Hld was identified as the most abundant component in *S. aureus* MVs of diverse genetic backgrounds. Hld overexpression enhanced the production of *S. aureus* MVs with relatively smaller sizes of MV particles, while Hld mutation or *agrA* deletion (*RNAIII* inactivation) induced differences in the composition of ^Δ*hld*^MVs and ^Δ*agrA*^MVs with increased protein diversity compared with that of ^wt^MVs. Loaded Hld could modify the properties of *S. aureus* MVs, including hemolytic activity, cytotoxicity, and inflammatory factor stimulation.

## 2. Materials and methods

### 2.1. Bacterial strains and culture conditions

Bacterial strains and plasmids used in this study are presented in Supplementary Table 1. The reference *S. aureus* strains RN4220 (NCTC 8325-4) were kindly provided by Prof. Baolin Sun (University of Science and Technology of China), USA300 (ATCC BAA-1556) was a gift from Dr. Min Li (Shanghai Jiao Tong University, China), Newman (NCTC 8178) was provided by Prof. Lu Yu (Jilin University, China), MW2 was a gift from Prof. Xiaoxue Ma (China Medical University), and strains ATCC29213, ATCC4330, and ATCC25923 and two clinical isolates (TJ274 and TJ275) were kindly provided by Dr. Dongmei Wang (The 983 Hospital of PLA). *S. aureus* XQ was isolated from the blood sample of a 16-year-old juvenile, whose onset was a skin wound then deteriorated to a lethal *S. aureus* sepsis with mental disorder (Rao et al., 2015; Liu et al., 2018). *S. aureus* strains were grown in brain heart infusion (BHI) medium (Oxoid, UK) at 37°C with shaking (200 rpm) or cultivated on BHI agar. *Escherichia coli* DH5α was cultivated in Luria–Bertani (LB) medium (Oxoid, UK) at 37°C with shaking or cultured on LB agar. For plasmid pBT2 (Supplementary Table 1) maintenance, cultures were supplemented with 50 μg/ml of ampicillin (AMP) and 10 μg/ml of chloramphenicol (Cm) for *E. coli* and *S. aureus*, respectively. All strains were stored in an appropriate medium with 20% glycerol at −80°C and resuscitated by streaking on agar plates. A single colony was picked and cultured with BHI or LB medium for the subsequent experiments.

### 2.2. Preparation of *S. aureus* MVs

MV isolation was performed with some modifications as previously described (Hong et al., 2011; Yuan et al., 2018; Chen et al., 2020; Zaborowska et al., 2021). In brief, a single colony of *S. aureus* RN4220 from the BHI agar plate was inoculated into 1 ml BHI medium and cultured at 37°C for 6 h. Then, 100 μl of culture was transferred to 10 ml BHI broth (1:100) and cultured at 37°C overnight with shaking. On the next day, the culture was inoculated into 900 ml BHI medium (1:100) and cultured at 37°C for 22 h to achieve the highest MV production (Yuan et al., 2018). The culture supernatant was collected after centrifugation at 6,000 × g at 4°C for 20 min to remove bacterial cells and centrifugation at 10,000 × g at 4°C for 30 min to remove cell debris and then filtered through a 0.45 μm vacuum filter (Millipore, USA), to remove possible cell debris or aggregates. The filtrate was concentrated to 240 ml with a 100 kDa cutoff hollow fiber cartridge column (GE Healthcare, USA) and then filtered through a 0.22 μm filter (Millipore, USA). The supernatant was ultracentrifuged at 200,000 × g at 4°C for 3 h.

The MV pellets were washed twice with phosphate-buffered saline (PBS, pH7.2) and resuspended in sterile PBS, and the amount of protein in the MVs was measured with Bradford Protein Assay Kit (Beyotime, China) as previously described (MacDonald and Beveridge, 2002; Tartaglia et al., 2018; Bitto et al., 2021b). Prepared MVs were stored at −80°C for use.

### 2.3. 4D-label-free liquid chromatography–tandem mass spectrometry analysis

The protein composition in MVs of *S. aureus* RN4220 (^wt^MVs) was identified by using 4D-label-free liquid chromatography–tandem mass spectrometry (LC-MS/MS) as previously described (Cox et al., 2014). In brief, the proteins in three biological replicate ^wt^MV samples were digested with trypsin (Promega, USA), desalted on a C18 cartridge column (Empore™, Sigma, USA), and reconstituted in 40 μl of 0.1% (v/v) formic acid. The peptides were subjected to 4D-label-free LC-MS/MS analysis by Applied Protein Technology (Shanghai, China). The MS raw data for each sample were searched against the database of *S. aureus* (http://www.uniprot.org/) by using MaxQuant 1.5.3.17 software (Parianichnikov et al., 2020). Cluster 3.0 and Java TreeView software were used to perform hierarchical clustering analysis (Hoon et al., 2004; Saldanha, 2004). CELLO (http://cello.life.nctu.edu.tw/) was used to predict protein subcellular localization. Protein sequences were searched using InterProScan software to identify protein domain signatures from the InterPro member database Pfam (Quevillon et al., 2005). The gene ontology (GO) was analyzed using the protein sequences of the selected differentially expressed proteins. NCBI BLAST+ client software and InterProScan were used to find homolog sequences; the GO terms were mapped, and sequences were annotated using software Blast2GO (Conesa et al., 2005; Quevillon et al., 2005; Camacho et al., 2009). The results of GO annotation were plotted by R scripts. Kyoto Encyclopedia of Genes and Genomes (KEGG) was analyzed using the online database (http://geneontology.org/). KEGG orthology identifications were retrieved and subsequently mapped to pathways in KEGG. Enrichment analysis was applied based on Fisher's exact test, considering the whole quantified proteins as the background dataset. Benjamini–Hochberg correction for multiple testing was further applied to adjust derived *P*-values. Only functional categories and pathways with *P*-values under a threshold of 0.05 were considered significant. All raw data of 4D label-free LC-MS/MS identification was deposited to the ProteomeXchange Consortium (http://proteomecentral.proteomexchange.org) via the iProX partner repository with the dataset identifier PXD039734.

### 2.4. SDS-PAGE and Western blot analysis

The purified MVs were mixed with 5 × SDS-PAGE loading buffer at a rate of 4:1 and incubated in boiling water for 10 min. The proteins in samples (10 μl/each) were separated by 12% (m/v) sodium dodecyl sulfate polyacrylamide gel electrophoresis (SDS-PAGE) and stained with Coomassie brilliant blue (Thermo Scientific, USA). For Western blot, the proteins after SDS-PAGE were transferred to a polyvinylidene difluoride (PVDF) membrane (GE Healthcare, USA). The PVDF membrane was blocked with PBS-T (PBS + 0.1% (v/v) Tween-20) containing 5% (m/v) skim milk (ABgent, USA) at 37°C for 60 min, followed by incubation with anti-Hld rabbit polyclonal primary antibody (1:1,000 dilution in PBS-T containing 5% (m/v) skim milk) at 4°C overnight. After five times of wash with PBS-T, the membrane was incubated with horseradish peroxidase-conjugated goat anti-rabbit secondary antibody (Abmart, China; 1:5,000 dilution) at 37°C for 1 h. After five times of wash with PBS-T, the target protein was visualized with the enhanced chemiluminescence system (Thermo Scientific) and photographed.

### 2.5. Gene knockout and genetic complementation

The site mutation *S. aureus* RN4220-Δhld and *agrA* deletion RN4220-ΔagrA mutants were generated via allelic replacement as previously described (Peng et al., 2017). All primers used in this study are presented in Supplementary Table 2. For the construction of RN4220-Δhld with normal RNAIII function, the 20th base “C” in the *hld* gene was changed to “G” to achieve a stop codon TGA and result in a truncated translation of Hld. In brief, the homologous left arm and homologous right arm were amplified with primer pairs delta-L/pBT2-delta-R (*Bam*HI) and pBT2-delta-L (*Sal*I)/delta-R, respectively, from the genomic DNA template of *S. aureus* RN4220 (GenBank accession no. NZ_CP101124) by polymerase chain reaction (PCR). The PCR fragments were purified and ligated to pBT2 plasmid via ClonExpress MultiS One Step Cloning Kit (Vazyme, China). The recombinant plasmid pBT2-Δhld was transformed into competent *E. coli* DH5α, extracted, and sequenced. The correct plasmid was then electrotransformed into *S. aureus* RN4220. The plasmid-carrying *S. aureus* was cultured at 30°C for 16 h, and the plasmid pBT2-Δhld was verified by double digestion with *Bam*HI and *Sal*I (TakaRa, Japan). The correct strain carrying pBT2-Δhld was inoculated into fresh BHI medium and cultivated at 42°C to induce the integration of the recombinant plasmid into the genome, which was verified by colony PCR with primer pairs delta-outer-L/pBT2-down and pBT2-up/delta-outer-R. The plasmid was eliminated via culturing a single colony at 25°C to achieve the mutant strain RN4220-Δhld, which was verified by PCR amplification with primer pair delta-outer-L/delta-outer-R and DNA sequencing. The construction of RN4220-ΔagrA used similar strategies mentioned above.

For genetic complementation, the tetracycline promoter *tetP* was introduced in front of the *hld* gene to obtain tetP/hld, which was inserted behind the *eno* gene on the genome of RN4220-Δhld. The homologous left arm, right arm, and *hld* gene were amplified by PCR with primer pairs up-pBT2-eno-tetP-L/up-tetP-R, down-eno-L/down-pBT2-eno-R, and tetP-hld-L/tetP-hld-R, respectively, from genomic DNA template of *S. aureus* RN4220. The *tetP* promotor fragment was amplified with primer pair tetP-L/tetP-R from the plasmid pYT3 (Supplementary Table 1). The four PCR fragments were purified and ligated to the pBT2 plasmid via ClonExpress MultiS One Step Cloning Kit. The resulting pBT2-tetP/hld was transformed into *S. aureus* RN4220-Δhld, and the genetic complementation strain RN4220-Δhld-tetP/hld was screened and identified using similar strategies for RN4220-Δhld construction. The *hld* overexpression strain RN4220-tetP/hld was generated after the transformation of pBT2-tetP/hld into the wild-type *S. aureus* RN4220.

### 2.6. Growth curve determination

The growth curves of *S. aureus* RN4220 and its derivatives RN4220-Δhld and RN4220-ΔagrA were determined. In brief, *S. aureus* strains of interest were cultured in BHI broth at 37°C with shaking overnight, and 0.2 ml of each strain culture was inoculated into 20 ml of fresh BHI medium. The optical density (OD) at 600 nm (OD600) was detected every hour for 15 h after inoculation. Growth curves were drawn using the OD600 values over culture times.

### 2.7. MV size evaluation

The size distribution of *S. aureus* MVs was evaluated by dynamic light scattering (DLS) analysis using Zetasizer Nano ZS90 (Malvern, UK). In brief, purified MVs were diluted in PBS to 0.1 mg/ml. Reads of 60-s durations were conducted in triplicate at 25°C. Data outputs were generated using Zetasizer (Nano, μV, APS) software v7.13 (Malvern, UK). The average number of particles at each binned center in the Experiment Summary output was adjusted by the dilution factor using GraphPad Prism 8.0.1 software, and the mean of three biological replicates was plotted as particles.

### 2.8. Hemolysis assay

A hemolysis assay was performed as previously described (Miller et al., 2021). Approximately 10 ml of whole blood was collected from the central auricular artery of a New Zealand male rabbit, which was purchased from the Laboratory Animal Center of the Third Military Medical University, and added to 1 ml heparin sodium solution, followed by three times of wash with PBS with centrifugation at 1,000 × g for 5 min at 4°C. A concentration of 2% (v/v) rabbit red blood cells (RBCs) was prepared with PBS. In total, 40 μl of PBS containing 2, 5, 10, and 20 μl, respectively, of 0.5 mg/ml MVs derived from *S. aureus* RN4220 (^wt^MVs) or its derivatives RN4220-Δhld (^Δ*hld*^MVs) and RN4220-ΔagrA (^Δ*agrA*^MVs) was added to 1 ml of 2% RBCs to the final concentration of 1, 2.5, 5, and 10 μg/ml of MVs, respectively, mixed gently, and incubated at 37°C for 30 min. After centrifuging at 8,000 × g for 10 s, the supernatant was collected, and OD540 values were determined with a spectrophotometer (Thermo Scientific). PBS-treated RBCs served as negative control, while RBC treatment with 1% (v/v) Triton X-100 represented positive control. The percentage hemolysis is calculated as follows: [(OD540 of sample−OD540 of negative control)/(D540 of positive control–OD540 of negative control)] × 100%.

### 2.9. Cell culture and stimulation

Macrophage RAW264.7 cells were purchased from a Chinese company Cas9X, maintained in Roswell Park Memorial Institute (RPMI) 1640 medium (Gibco, USA), and supplemented with 10% (v/v) fetal bovine serum (Zeta Life, USA) and 1% (v/v) penicillin/streptomycin (Beyotime, China) at 37°C in a humidified 5% CO_2_ atmosphere. RAW264.7 cells were seeded at 2.5 × 10^5^ cells/ml in 500 μl in 24-well plates for 12 h and stimulated with 5, 10, and 20 μg/ml of ^wt^MVs, ^Δ*hld*^MVs, or ^Δ*agrA*^MVs for 6 h, and the cell culture supernatants were collected for further study.

### 2.10. Cell viability assay and lactate dehydrogenase release detection

The cell viability was detected by a CCK-8 cell viability kit (ABclonal, China), according to the instructions provided by the manufacturer. In brief, RAW264.7 cells were seeded at 2.5 × 10^5^ cells/ml in 100 μl RPMI1640 medium in 96-well plates for 12 h and stimulated with 10 μl of 0.05, 0.1, and 0.2 mg/ml of ^wt^MVs, ^Δ*hld*^MVs, or ^Δ*agrA*^MVs (the final concentration of 5, 10, and 20 μg/mL of MVs, respectively) for 6 h at 37°C with 5% CO_2_. In total, 10 μl of CCK-8 solution was added to each well and incubated for 2 h, and OD450 values were determined with a spectrophotometer (Thermo Scientific). The 10 μl of PBS-treated RAW264.7 served as a negative control, and the wells with culture medium and CCK-8 solution served as blank controls. The percentage of cell-survival rate is calculated as follows: [(OD450 of sample−OD450 of blank control)/(OD450 of negative control−OD450 of blank control)] × 100%.

The LDH cytotoxicity assay kit (Beyotime, China) was used to quantify the activity of LDH released from damaged cells. RAW264.7 cells were seeded at 1 × 10^5^ cells/ml in 200 μl of RPMI1640 medium in 96-well plates for 12 h, the culture solution was discarded, and the cells were washed once with sterile PBS. In total, 200 μl of fresh low serum culture medium (1% serum) and 20 μl of PBS containing 0.05, 0.1, and 0.2 mg/ml of ^wt^MVs, ^Δ*hld*^MVs, or ^Δ*agrA*^MVs were added to each well to the final concentrations of 5, 10, and 20 μg/ml of MVs, respectively. Cell-free culture medium supplemented well served as blank control, untreated cell-seeded well served as sample control, 20 μl PBS-treated RAW264.7 served as negative control, and cells lysed with 20 μl LDH releasing reagent used as maximum enzyme activity control. After 6 h of incubation, the plate was centrifuged at 400 × g for 5 min, and 120 μl supernatant of each well was transferred to a new 96-well plate, followed by adding 60 μl of detection working solution, mixed well, and incubated at room temperature to avoid light for 30 min. OD490 and OD600 values were determined. The measured OD490 and OD600 values were each subtracted from the values of blank control. LDH activity is calculated as follows: [((OD490 − OD600 of sample) − (OD490 − OD600 of sample control))/((OD490 − OD600 of maximum enzyme activity control) − (OD490 − OD600 of sample control))] × 100%.

### 2.11. Detection of inflammatory factors

All animal study was conducted in accordance with the ARRIVE guidelines. In total, 6- to 8-week-old female BALB/c mice (20 ± 2 g) were purchased from Hunan Slyke Jingda Experimental Animal Co. LTD. All animal experiments were approved by the Laboratory Animal Welfare and Ethics Committee of the Army Medical University (protocol No. AMUWEC2020735).

BALB/c mice (*n* = 3) were intraperitoneally injected 100 μl (10 or 20 μg/ml) of ^wt^MVs, ^Δ*hld*^MVs, or ^Δ*agrA*^MVs. PBS was used as the control. Mouse sera were collected 6 h post-injection. Then, the levels of inflammatory factors, including IL-6, IL-1β, and TNFα, were determined with an enzyme-linked immunosorbent assay (ELISA) kit, according to the manufacturer's instructions (ABclonal, China). The results were examined with an ELISA reader Multiskan Go (Thermo Scientific), and inflammatory factor levels were determined according to the standard curve.

### 2.12. Statistical analysis

All statistical analyses were performed using GraphPad Prism 8.0.1 software. One-way analysis of covariance (ANOVA) was used for comparing multiple groups. The results were presented as mean ± standard deviation (SD), and *P*-values of <0.05 were considered to be statistically significant.

## 3. Results

### 3.1. Abundant Hld loaded in the MVs of *S. aureus* with diverse genetic lineages

**Figure 1:**
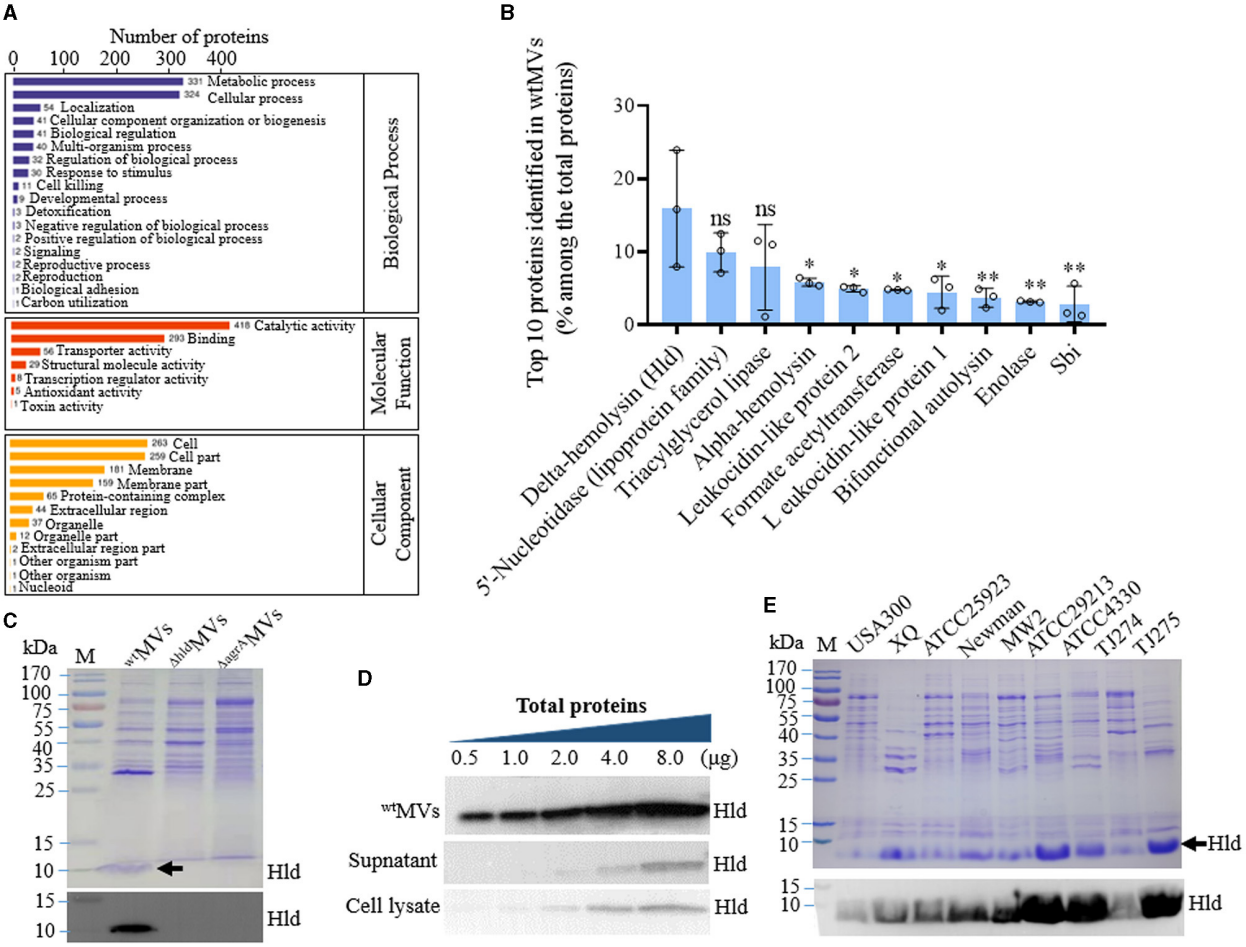
Hld loaded in the MVs of *S. aureus* with diverse genetic lineages. **(A)** GO function analysis of proteins identified with 4D-label-free LC-MS/MS in the MVs of *S. aureus* RN4220 (^wt^MVs). Proteins involved in representative biological processes, molecular functions, and cellular components were indicated. **(B)** Proportion of the top 10 proteins identified in ^wt^MVs counted by label-free quantity (LFQ) intensity. The data were presented as mean ± SD of three biological replicates. Statistical significance was calculated by one-way ANOVA and compared with the LFQ intensity of Hld; ns indicates no statistical significance; ^∗^*P* < 0.05 and ^∗∗^*P*< 0.01. **(C)** Characterization of Hld in the MVs of *S. aureus* RN4220 and its derivatives. Comparison of protein profiles among ^wt^MVs, ^Δ*hld*^MVs derived from RN4220-Δhld, and ^Δ*agrA*^MVs prepared from RN4220-ΔagrA by SDS-PAGE (top panel), the loaded Hld was indicated by a black arrow. Identification of loaded Hld in ^wt^MVs, ^Δ*hld*^MVs, and ^Δ*agrA*^MVs by Western blot (bottom panel). **(D)** Western blot analysis of Hld in ^wt^MVs, culture supernatant, and cell lysate of RN4220. Diverse total proteins in the samples were subjected to Hld detection and loaded Hld presented in the ^wt^MVs. **(E)** Detection of loaded Hld in the MVs derived from *S. aureus* strains with different genetic lineages. SDS-PAGE analysis of protein profiles of MVs from indicated *S. aureus* strains and the loaded Hld in each bacterial MV was shown by a black arrow (top panel). Detection of loaded Hld in each bacterial MVs by Western blot (bottom panel).

An accurate proteome-wide 4D-label-free LC-MS/MS (Cox et al., 2014) was performed to quantify the proteins in MVs of *S. aureus* RN4220 (^wt^MVs). A total of 704 proteins in the ^wt^MVs were identified, and they were predicted to be membrane proteins (178, 25.3%), extracellular (147, 20.9%), and/or cytoplasmic proteins (458, 65.1% with some overlapped ones). KEGG enrichment revealed that oxidative phosphorylation was the most significant pathway involved, followed by citrate cycle, glycolysis/gluconeogenesis, pyruvate metabolism, carbon-fixation pathways in prokaryotes, and nucleotide metabolism (Supplementary Figure 1). GO analysis showed that the enriched proteins were involved in metabolic and cellular processes, catalytic activity and binding of molecular functions, and cell and cell part of cellular components (Figure 1A).

Notably, Hld was the most abundant molecule in ^wt^MVs of *S. aureus* RN4220, which presented a label-free quantity (LFQ) intensity of 367,430,000 and accounted for 15.6 ± 7.9% of the total protein quantity identified (Figure 1B). SDS-PAGE analysis also revealed the loaded Hld in ^wt^MVs, which could be characterized by specific antibodies against Hld in Western blot assay (Figure 1C; Supplementary Figure 2). Compared with the protein in bacterial cell lysate and culture supernatant, Hld was remarkably loaded in the ^wt^MVs of *S. aureus* RN4220 and elevated in an MV-dose-dependent manner (Figure 1D; Supplementary Figure 2). To determine whether loaded Hld occurred in MVs derived from other strains, we cultured nine *S. aureus* strains (USA300, XQ, ATCC25923, Newman, MW2, ATCC29213, ATCC4330, TJ274, and TJ275) and individually prepared their MVs with the same conditions to RN4220. SDS-PAGE and Western blot assays revealed the loaded Hld in all MVs tested (Figure 1E; Supplementary Figure 2), suggesting that the encapsulation of Hld in *S. aureus* MVs is common.

### 3.2. Hld promoted the production of *S. aureus* MVs with decreased size

**Figure 2:**
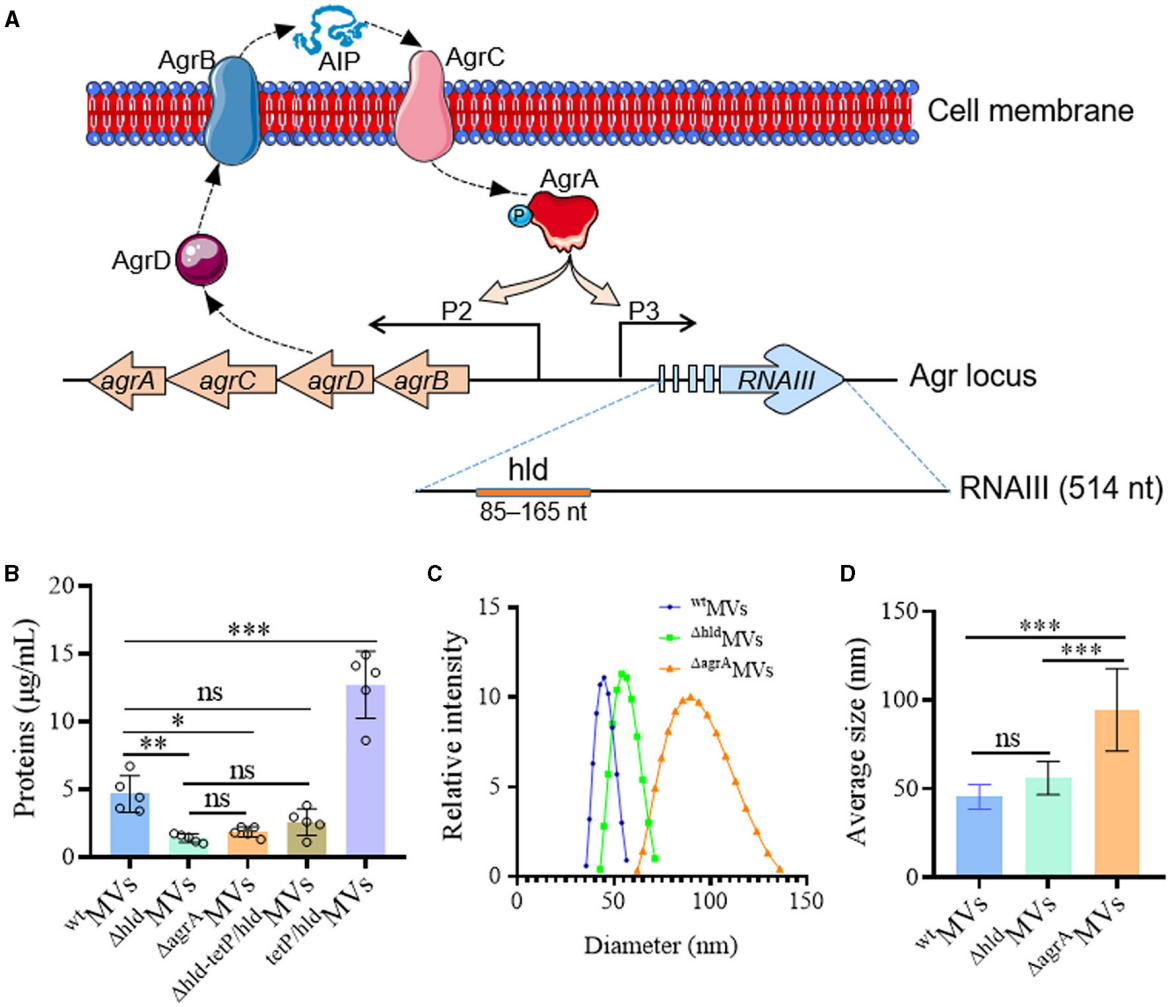
Hld promoted the production of *S. aureus* MVs with smaller sizes. **(A)** Schematic diagram showing the *agr* system and the *hld* gene located in RNAIII. AgrD is processed by AgrB to generate auto-inducing peptide (AIP), which can bind AgrC to activate AgrA by phosphorylation. AgrA can not only stimulate P2 promotor to promote Agr function but also activate P3 promotor to produce effective regular RNAIII, which guides Hld expression. **(B)** Hld enhanced MV production in *S. aureus* RN4220. The proteins in ^wt^MVs (derived from RN4220), ^Δ*hld*^MVs (from RN4220-Δhld), ^Δ*agrA*^MVs (from RN4220-ΔagrA), ^Δ*hld*−*telP*/*hld*^MVs (prepared from complementation strain RN4220-Δhld-tetP/hld), and ^telP/hld^MVs (derived from overexpression strain RN4220-tetP/hld) were quantified by Bradford assay. The data were presented as mean ± SD (*n* = 5). **(C)** The size distribution and **(D)** the sizes of ^wt^MVs, ^Δ*hld*^MVs, and ^Δ*agrA*^MVs measured by DLS. The data were presented as mean ± SD (*n* = 3). Statistical significance was calculated by one-way ANOVA; ns indicates no statistical significance; ^∗^*P* < 0.05, ^∗∗^*P* < 0.01, and ^∗∗∗^*P* < 0.001.

The gene encoding Hld is within the RNAIII molecule (85–165 nt) (Figure 2A), a global RNA regulator involved in the *S. aureus* Agr quorum-sensing system (Hodille et al., 2016). To evaluate the function of loaded Hld in MVs, we generated an *S. aureus* mutant (RN4220-Δhld) by changing the 20th base of C to G in the *hld* gene to achieve a stop codon TGA and abolish the translation of Hld protein (Supplementary Figures 3A, B). An *agrA* gene knocked-out strain (RN4220-ΔagrA) was also constructed to inactivate RNAIII and Hld functions (Supplementary Figure 3C). Compared with wild-type RN4220, *agrA* deletion and site mutation in the *hld* gene did not affect the growth of RN4220-Δhld and RN4220-ΔagrA mutants (Supplementary Figure 3D). No Hld was detected in ^Δ*hld*^MVs and ^Δ*agrA*^MVs derived from RN4220-Δhld and RN4220-ΔagrA, respectively (Figure 1C). Western blot showed that the expressions of virulence factors such as alpha-hemolysin (Hla), gamma-hemolysin B subunit (HlgB), and Panton–Valentine leukocidin (PVL) element LukS in the RN4220-Δhld were similar to those in the wild-type strain (Supplementary Figures 3E, F). However, *agrA* deletion resulted in decreased expression of all virulence factors tested. These data indicated that the site-mutation strain RN4220-Δhld disabled Hld expression but had a functional RNAIII, which controlled the expression of many virulence factors, including Hla, HlgB, and PVL-LukS. By contrast, Hld expression and RNAIII function were absent in the RN4220-ΔagrA.

The MV production in RN4220-Δhld and RN4220-ΔagrA was substantially reduced compared with that in wild-type RN4220 (Figure 2B). For complementation, a tetracycline promoter TetP-controlled *hld* gene was constructed in RN4220-Δhld by inserting it behind the *eno* gene, which encoded the MV-associated enolase (Yuan et al., 2018). The restored MV production was observed in RN4220-Δhld-tetP/hld (Figure 2B). Moreover, Hld overexpression in wild-type strain RN4220 (RN4220-tetP/hld) significantly increased MV yield (*P* < 0.001; Figure 2B). Next, the sizes of ^wt^MVs, ^Δ*hld*^MVs, and ^Δ*agrA*^MVs were measured, and the results showed that the sizes of ^wt^MVs (45.4 ± 6.9 nm), ^Δ*hld*^MVs (56.0 ± 9.3 nm), and ^Δ*agrA*^MVs (94.5 ± 23.2 nm) enlarged gradually (Figure 2C). The ^Δ*agrA*^MVs presented the largest particle size, which was significantly larger than those derived from RN4220-Δhld and wild-type RN4220 (*P* < 0.001; Figure 2D). These data suggested that Hld promoted MV production in *S. aureus*. Loaded Hld was associated with smaller MV particles, and other Agr-regulated factors may synergistically condense the sizes of *S. aureus* MVs.

### 3.3. Hld reduced the diversity of MV-loaded proteins

**Figure 3:**
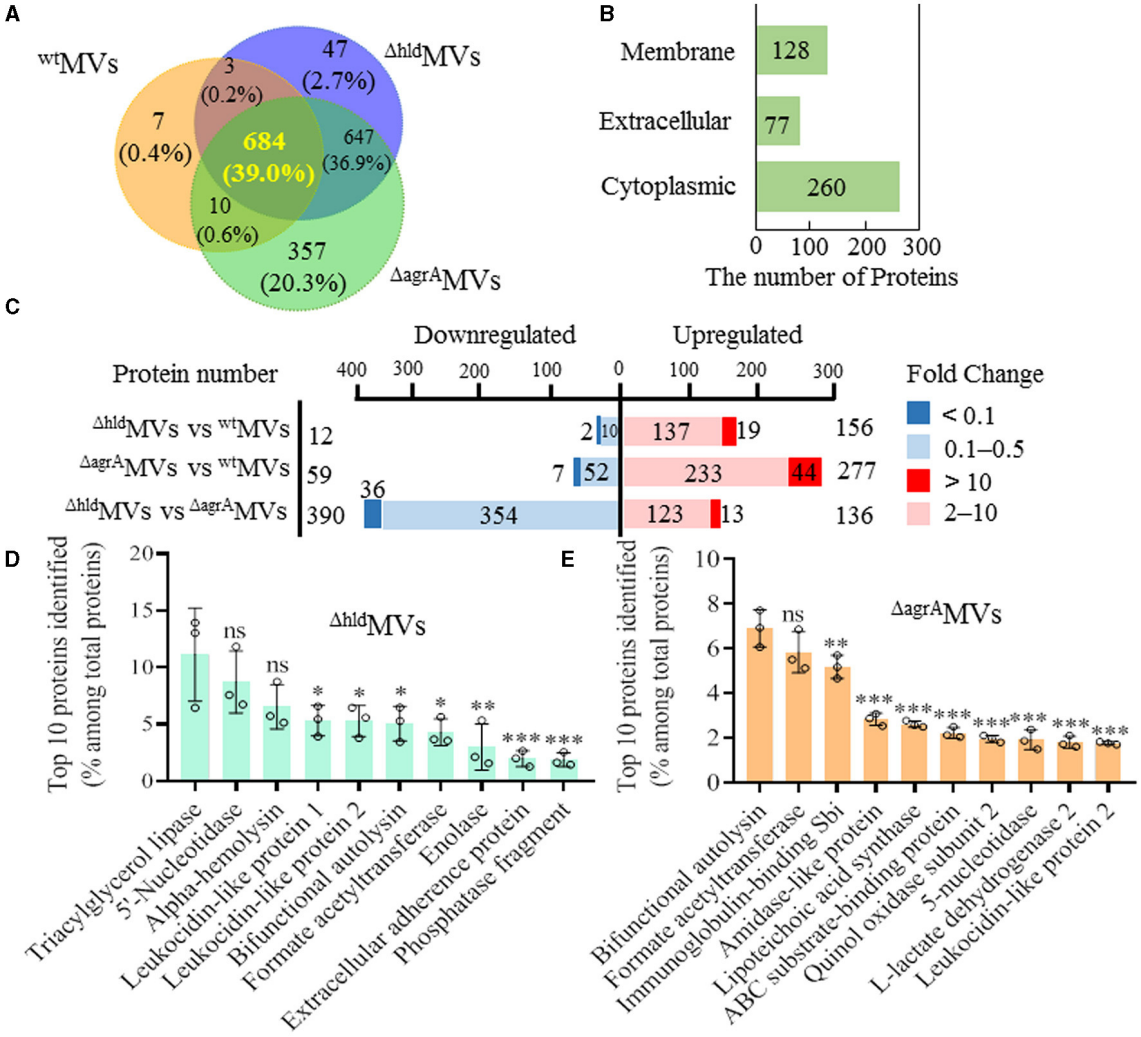
Comparative proteomic analysis of MVs from *S. aureus* RN4220, RN4220-Δhld, and RN4220-ΔagrA. **(A)** Venn diagram showing protein overlap among ^wt^MVs, ^Δ*hld*^MVs, and ^Δ*agrA*^MVs. **(B)** Subcellular localization of differentially expressed proteins in ^Δ*hld*^MVs and ^Δ*agrA*^MVs. **(C)** Differentially expressed proteins between paired MVs indicated. The fold change ≥2 and ≤0.5 (*P* < 0.05) were considered as significantly upregulated and downregulated proteins, respectively. The protein number with fold change >10 or <0.1 was separately indicated. The top 10 proteins identified in **(D)** ^Δ*hld*^MVs and **(E)** ^Δ*agrA*^MVs. The proportion of the predominant proteins in MVs was characterized by LFQ intensity. The data were presented as mean ± SD (*n* = 3). Statistical significance was calculated by one-way ANOVA and compared with the LFQ intensity of the top one protein; ns indicates no statistical significance; ^∗^*P* < 0.05, ^∗∗^*P* < 0.01, and ^∗∗∗^*P* < 0.001.

RN4220-Δhld and RN4220-ΔagrA showed lower abilities to produce MVs compared with wild-type RN4220. 4D-label-free proteomics was further performed to detect differentially loaded proteins in the ^Δ*hld*^MVs and ^Δ*agrA*^MVs. The results showed that 684 proteins were shared in ^wt^MVs, ^Δ*hld*^MVs, and ^Δ*agrA*^MVs (Figure 3A). Compared with proteins included in ^wt^MVs (704), Hld mutation resulted in increased proteins encapsulated in ^Δ*hld*^MVs (1,381), and RNAIII inactivation even enhanced the proteins loaded by ^Δ*agrA*^MVs (1,698). The differentially loaded proteins in ^wt^MVs, ^Δ*hld*^MVs, and ^Δ*agrA*^MVs were primarily predicted cytoplasmic proteins (260), extracellular components (77), and membrane-associated proteins (128, Figure 3B). Compared with ^wt^MVs, ^Δ*hld*^MVs had 156 upregulated (fold change ≥ 2) and 12 downregulated (fold change ≤ 0.5) proteins, whereas ^Δ*agrA*^MVs exhibited 277 upregulated and 59 downregulated proteins. However, ^Δ*hld*^MVs presented 136 upregulated and 390 downregulated proteins relative to ^Δ*agrA*^MVs (Figure 3C). These data indicated that loaded Hld was associated with reduced protein diversity in *S. aureus* MVs.

The top 10 MV proteins dominant in ^Δ*hld*^MVs and ^Δ*agrA*^MVs were analyzed. Out of the top 10 proteins, 8 were shared by ^Δ*hld*^MVs and ^wt^MVs (Figures 1B, 3D). Among the top 10 MV proteins, Hld and immunoglobulin-binding protein Sbi were unique in ^wt^MVs, whereas extracellular adherence protein and 30 kDa neutral phosphatase fragment were specific for ^Δ*hld*^MVs. By contrast, ^Δ*agrA*^MVs had diverse abundant proteins (Figures 1B, 3E). KEGG analysis revealed that the upregulated proteins in ^Δ*hld*^MVs vs. ^wt^MVs were involved in ABC transporters, bacterial secretion, photosynthesis, and protein export, whereas the downregulated proteins were enriched in purine metabolism, quorum sensing, biosynthesis of cofactors, and nucleotide metabolism (Supplementary Figure 4A). GO analysis indicated that the most abundant differentially loaded proteins in ^Δ*hld*^MVs vs. ^wt^MVs were involved in cellular metabolic process and localization for biological process; catalytic activity, binding, and transporter activity for molecular function; and membrane, membrane part, cell, and cell part for cellular component (Supplementary Figure 4B). These results demonstrated that the loading of Hld in ^wt^MVs may inhibit the encapsulation of other proteins and affect the diversity of MV-loaded proteins.

### 3.4. Loaded Hld contributed to the hemolytic activity of *S. aureus* MVs

**Figure 4:**
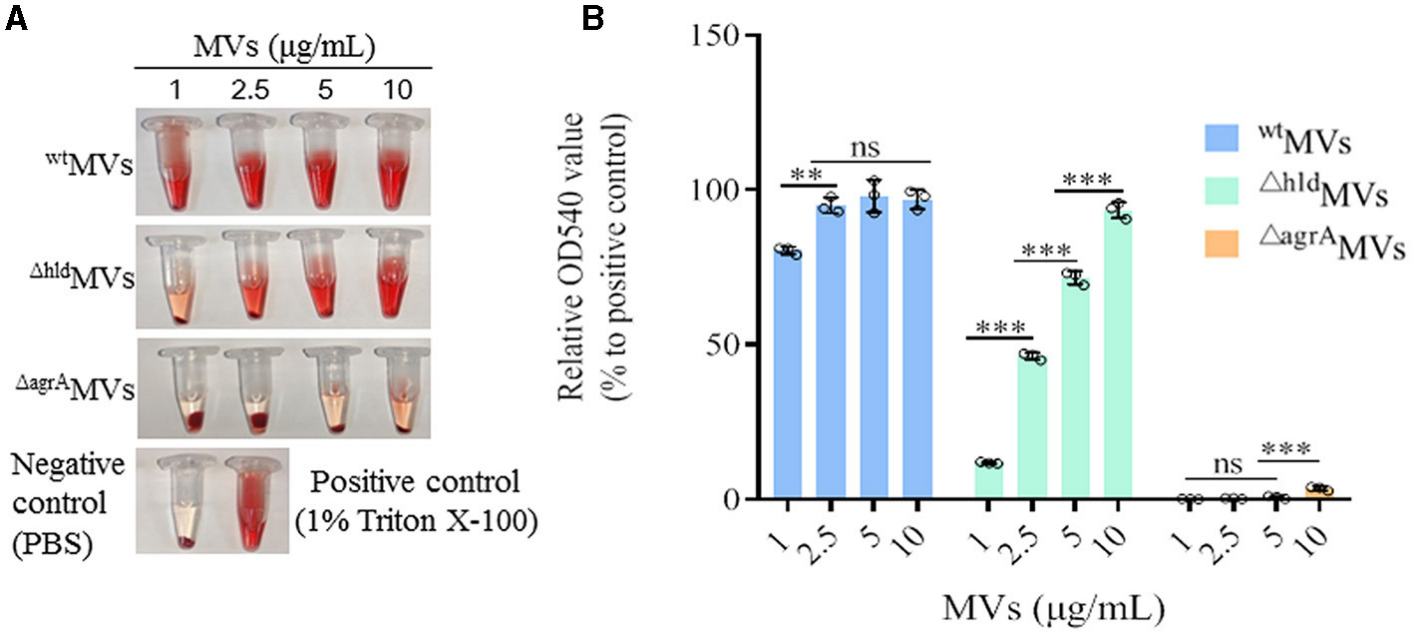
Hemolytic activity of ^wt^MVs, ^Δ*hld*^MVs, and ^Δ*agr*^AMVs. **(A)** Hemolysis tested in tubes. Representative tubes carried 2% rabbit red blood cells after treatment with diverse concentrations of ^wt^MVs, ^Δ*hld*^MVs, and ^Δ*agr*^AMVs. PBS-treated cells served as negative control, and 1% Triton X-100-treated cells served as positive control. **(B)** Hemolysis rate determination. The OD540 value of each hemolysis tube was determined. The OD540 value relative to the positive control was calculated. Data are presented as the mean ± SD of three independent experiments. Statistical significance was calculated by one-way ANOVA; ns represents no significance, ^∗∗^*P* < 0.01 and ^∗∗∗^*P* < 0.001.

Hld plays crucial roles in staphylococcal pathogenicity, such as causing hemolysis and allergy (Nakamura et al., 2013). The hemolytic activities of ^wt^MVs, ^Δ*hld*^MVs, and ^Δ*agrA*^MVs were detected. Rabbit RBCs (2%) were mixed with 1, 2.5, 5, and 10 μg/ml of MVs, respectively, and incubated at 37°C for 30 min. The values of OD540 were detected, and the results showed that ^wt^MVs presented higher hemolytic activity than ^Δ*hld*^MVs and ^Δ*agrA*^MVs (Figure 4A). The hemolytic activity of ^Δ*hld*^MVs prepared from Hld mutant RN4220-Δhld significantly increased in an MV-dose-dependent manner (*P* < 0.001, Figure 4B). However, different concentrations of ^Δ*agrA*^MVs derived from the *agrA* deletion strain RN4220-ΔagrA presented comparable hemolytic activity to PBS control. The content of hemolytic proteins in MVs was analyzed by proteomics, and the results showed that ^wt^MVs loaded a large amount of hemolytic proteins with the most Hld, which accounted for 15.9% of the total proteins in ^wt^MVs. The proportion of hemolytic proteins in ^Δ*hld*^MVs was reduced, and Hla occupied the most in ^Δ*hld*^MVs (6.8%). By contrast, the content of hemolytic proteins in ^Δ*agrA*^MVs was the lowest (Supplementary Figure 5). These results indicated that Hld was an important component that contributed to the hemolytic activity of ^wt^MVs, and other factors such as Hla and leukocidin-like proteins may also play important roles in the hemolytic activity of *S. aureus* MVs.

### 3.5. Hld-loaded MVs were cytotoxic to macrophages

**Figure 5:**
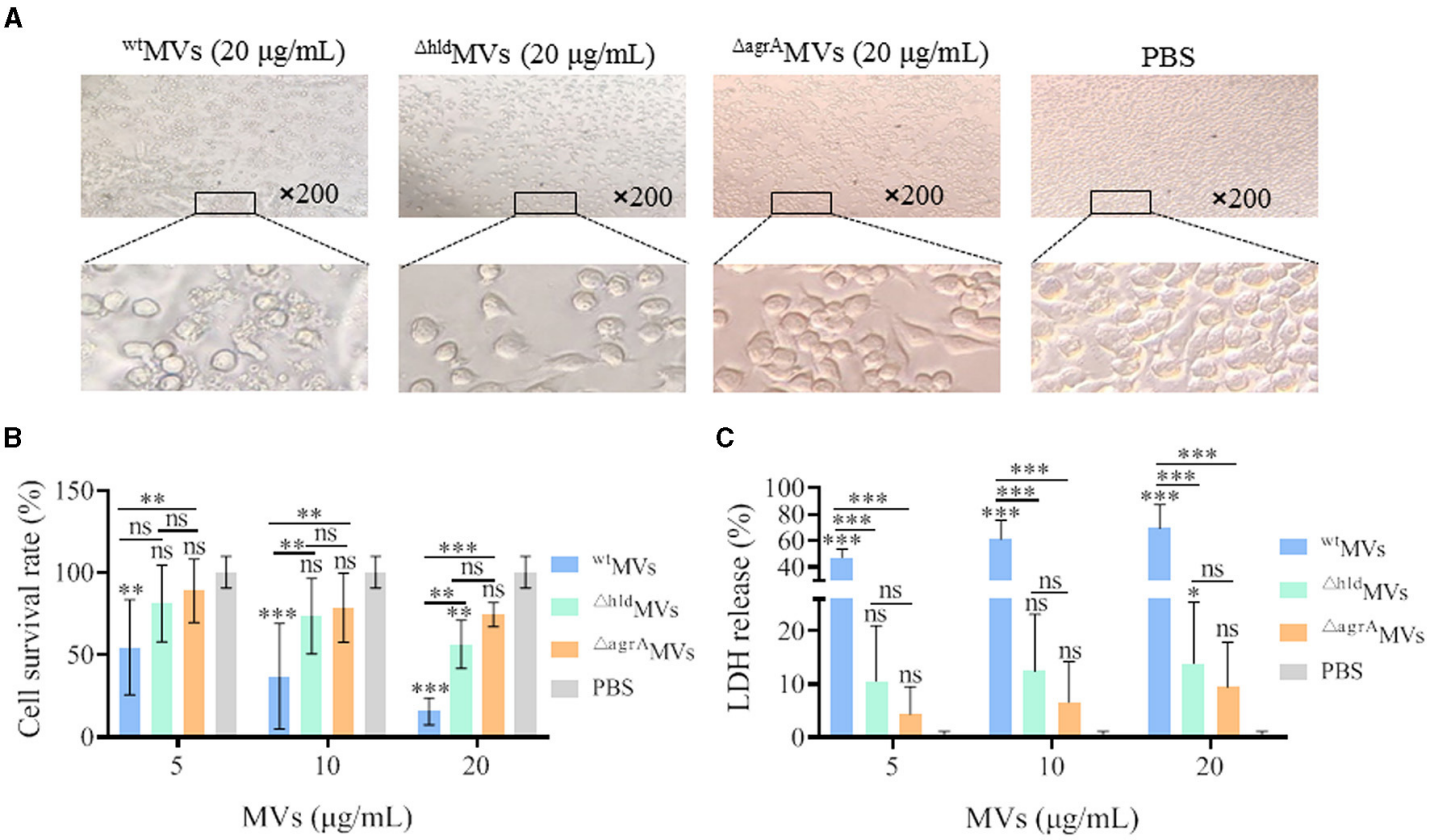
Effect of MVs on macrophage RAW264.7 cells. **(A)** Morphological changes of RAW264.7 cells after treatment with ^wt^MVs, ^Δ*hld*^MVs, and ^Δ^*agrA* MVs at 20 μg/ml concentration. **(B)** Cell-survival rate of RAW264.7 cells. Macrophage cells were treated with different concentrations of ^wt^MVs, ^Δ*hld*^MVs, or ^Δ*agrA*^MVs for 6 h, and the cell-survival rate was measured by CCK-8 assay. **(C)** LDH release of RAW264.7 cells. Cells were treated with diverse concentrations of ^wt^MVs, ^Δ*hld*^MVs, or ^Δ*agrA*^MVs for 6 h, and LDH release was measured by a Cytotoxicity Assay Kit. PBS served as a negative control. Data are presented as mean ± SD of three independent experiments. Statistical significance was calculated by one-way ANOVA relative to that of PBS control in each group, and the other comparisons were also indicated; ns represents no significance; ^∗^*P* < 0.1, ^∗∗^*P* < 0.01, and ^∗∗∗^*P* < 0.001.

Macrophage RAW264.7 cells were treated with 5, 10, and 20 μg/ml of ^wt^MVs, ^Δ*hld*^MVs, or ^Δ*agrA*^MVs for 6 h, and the cell death was evaluated. Microscopy revealed that RAW264.7 treated with 20 μg/ml of ^wt^MVs presented greater cell disruption than those treated with ^Δ*hld*^MVs (Figure 5A), whereas the morphology of RAW264.7 treated with 20 μg/ml ^Δ*agrA*^MVs was similar to that of the PBS control group. CCK-8 assay and LDH release detection demonstrated that ^wt^MV was highly toxic, even at the low concentration of 5 μg/ml (Figures 5B, C). However, the toxicity of ^Δ*hld*^MVs was significantly reduced, and only a high concentration of ^Δ*hld*^MVs (20 μg/ml) presented toxicity to RAW264.7 cells compared with the PBS control. No significant difference in cell survival and LDH release was observed between ^Δ*agrA*^MVs and PBS treatment. The proteomic data showed that ^wt^MVs loaded a large amount of virulence proteins (33.7%), whereas 19.36% of the total proteins in ^Δ*hld*^MVs were counted to be virulence factors, and only 5.1% of virulence proteins were loaded in ^Δ*agrA*^MVs (Supplementary Figure 5). These data suggested that Hld-loaded ^wt^MVs were more toxic to RAW264.7 macrophages.

### 3.6. Loaded Hld MVs promoted the production of inflammatory factors *in vivo*

**Figure 6:**
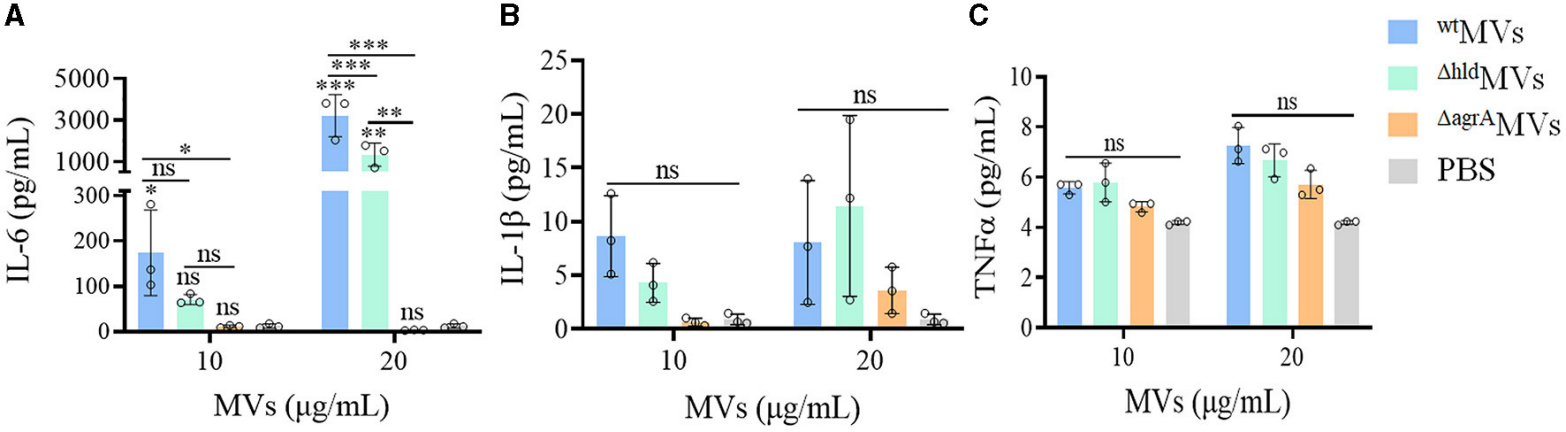
Effects of MVs on inflammatory factor production *in vivo*. Mice were injected with ^wt^MVs, ^Δ*hld*^MVs, or ^Δ*agrA*^MVs for 6 h, and the levels of inflammatory factors **(A)** IL-6, **(B)** IL-1β, and **(C)** TNFα in the mouse sera were determined with an ELISA kit. PBS served as a negative control. Data are presented as the mean ± SD (*n* = 3 for each group). Statistical significance was calculated by one-way ANOVA relative to that of PBS control in each group, and the other comparisons were also indicated; ns represents no significance, ^∗^*P* < 0.05, ^∗∗^*P* < 0.01, and ^∗∗∗^*P* < 0.001.

The levels of inflammatory factor production represent the activation of the immune system caused by bacterial virulence factors *in vivo*. BALB/c mice were intraperitoneally injected with different concentrations of ^wt^MVs, ^Δ*hld*^MVs, and ^Δ*agrA*^MVs (*n* = 3 per group). The sera were collected at 6 h post-injection and subjected to quantification of IL-6, IL-1β, and TNFα levels through ELISA. As shown in Figure 6A, the IL-6 levels in mice treated with 20 μg/ml of ^wt^MVs significantly increased compared with those in ^Δ*hld*^MV- and ^Δ*agrA*^MV-challenged mice (*P* < 0.001). However, comparable serum IL-1β and TNFα levels were presented between MV- and PBS-treated mice (Figures 6B, C). These results indicated that loaded Hld was a potent virulence factor for *S. aureus* MV pathogenicity.

## 4. Discussion

MV release is a ubiquitous process of bacteria during their growth *in vitro* and *in vivo* (Beveridge, 1999). The proteins identified in MVs vary due to bacterial species, strains, culture conditions, MV preparation processes, and characterization methods used (Bitto et al., 2021b). A total of 431 proteins have been identified from MVs of the gram-positive pathogen *Clostridium perfringens* strain CP4 by using 1D gel separation and LC-MS/MS analysis (Jiang et al., 2014). With label-free quantitative LC-MS/MS determination, 543 proteins are characterized from MVs of a clinical isolate *Cutibacterium acnes* (Jiang et al., 2022). Lee et al. (2009) first discovered MV release in *S. aureus* ATCC14458 and identified approximately 90 proteins in the MVs through nano-LC-ESI-MS/MS. A total of 222 proteins are consistently characterized in MVs derived from *S. aureus* N305, a bovine mastitis isolate (Tartaglia et al., 2018), and ~253 proteins are associated with MVs derived from *S. aureus* strains O11, O46, RF122, and MW2 after nano-LC-ESI-MS/MS analysis (Tartaglia et al., 2020). In the current study, 4D-label-free LC-MS/MS was used to identify proteins encapsulated in MVs of *S. aureus* RN4220 (Cox et al., 2011; Meier et al., 2018). A total of 704 proteins were consistently identified in all three parallel biological samples of ^wt^MVs derived from wild-type RN4220. The high sensitivity and deep coverage of 4D-label-free LC-MS/MS analysis resulted in 612 more proteins determined in ^wt^MVs derived from the same *S. aureus* strain (Yuan et al., 2018).

The function of certain MV-loaded proteins is seldom investigated, but the loading of some components such as cytoplasmic proteins and virulence factors has been characterized in bacterial MVs (Yuan et al., 2018). Herein, Hld presented the highest LFQ intensity in the 4D-label-free LC-MS/MS analysis of proteins loaded in ^wt^MVs (Figure 1B). SDS-PAGE and Western blot assays revealed that Hld was the most abundant protein in MVs prepared from *S. aureus* strains with different genetic lineages (Figures 1C, E), which was confirmed by another study (Jeon et al., 2016). Our data and other findings suggested that loaded Hld in staphylococcal MVs is a common feature. As one of the major virulence factors, Hld is involved in staphylococcal infections by targeting an extensive variety of mammalian cells, such as human monocytes, polymorphonuclear leukocytes, macrophages, mast cells, and erythrocytes (Hodille et al., 2016; Rao et al., 2022). MV-assisted release and transportation may be very important for Hld function. Our results showed that loaded Hld contributed to the hemolytic activity of *S. aureus* MVs, and Hld mutation remarkably reduced the hemolytic activity of ^Δ*hld*^MVs (Figure 4). Moreover, Hld-loaded ^wt^MVs were more cytotoxic to macrophage RAW264.7 cells than ^Δ*hld*^MVs and ^Δ*agrA*^MVs (Figures 5A, C). Importantly, Hld-loaded ^wt^MVs increased the stimulatory effect of the inflammatory factor production *in vivo* (Figure 6), indicating that Hld-loaded ^wt^MVs had strong virulence.

The mechanisms underlying *S. aureus* MV production and regulation are largely unknown (Qiao et al., 2021). The present study revealed that Hld played an important role in the production of *S. aureus* MVs (Figure 2B). In view of MV application, the large-scale preparation of bacterial MVs is valuable (Wang et al., 2018). However, Hld utilization in promoting staphylococcal MVs may raise safety concerns, such as Hld loading and its cytotoxicity. Interestingly, our data demonstrated that Hld promoted the production of *S. aureus* MVs with relatively smaller sizes, Hld mutation considerably enlarged the size of ^Δ*hld*^MVs, and Agr inactivation resulted in even larger ^Δ*agrA*^MVs (Figures 2C, D). These data indicated that Agr-associated virulence factors may reduce the size of *S. aureus* MV particles. Other factors involved in S. *aureus* MV size compression are worthy of further investigation.

Apart from the yield increase and size reduction of Hld-loaded ^wt^MVs, the proteins included in ^wt^MVs (704) were fewer than those encapsulated in ^Δ*hld*^MVs (1,381) and ^Δ*agrA*^MVs (1,698) after 4D-label-free LC-MS/MS analysis (Figure 3A). The encapsulation of Hld in ^wt^MVs decreased the diversity of proteins loaded in *S. aureus* MVs, but the reason for this phenomenon is unclear. Smaller sizes and a competitive mechanism may contribute to the encapsulation of certain proteins into *S. aureus* MVs. The top 10 MV proteins dominant in ^wt^MVs, ^Δ*hld*^MVs, and ^Δ*agrA*^MVs were analyzed (Figures 1B, 3D, E). Among the top 10 loaded proteins, Hld and immunoglobulin-binding protein Sbi were unique for ^wt^MVs (Al Kindi et al., 2021), whereas extracellular adherence protein and 30 kDa neutral phosphatase fragment dominated in ^Δ*hld*^MVs. KEGG analysis showed that upregulated proteins in ^Δ*hld*^MVs vs. ^wt^MVs primarily functioned in ABC transporters, bacterial secretion, and protein export, whereas the downregulated ones were enriched in purine metabolism, quorum sensing, and nucleotide metabolism (Supplementary Figure 4A). These results suggested that the inhibition of protein loading into *S. aureus* MVs by Hld may be selective, with a decrease in transport-related proteins. The significance and mechanism underlying the Hld modification of *S. aureus* MV protein encapsulation require further investigation.

In conclusion, our data shed light on the role of Hld in the property modification of *S. aureus* MVs. Hld was identified as the most abundant toxin in MVs of *S. aureus* strains with diverse genetic lineages. It could promote the release of staphylococcal MVs with smaller sizes than those derived from Hld mutant. Moreover, loaded Hld resulted in reduced protein diversity in *S. aureus* MVs. The loaded Hld contributed to the hemolytic activity of ^wt^MVs, which exhibited greater toxicity to macrophage RAW264.7 cells than ^Δ*hld*^MVs and ^Δ*agrA*^MVs. Furthermore, Hld-loaded ^wt^MVs promoted the production of inflammatory factor IL-6 *in vivo*. The loading and multifaceted roles of Hld in the vesicle property modification of *S. aureus* MVs suggested that a rational construction was needed to prepare MVs for application.

## Data availability statement

The datasets presented in this study can be found in online repositories. The names of the repository/repositories and accession number(s) can be found in the article/Supplementary material.

## Ethics statement

The animal study was approved by Laboratory Animal Welfare and Ethics Committee of the Army Medical University (Protocol No. AMUWEC2020735). The study was conducted in accordance with the local legislation and institutional requirements.

## Author contributions

XR: Conceptualization, Project administration, Supervision, Writing—review and editing. JC: Funding acquisition, Data curation, Investigation, Methodology, Software, Writing—original draft. YL: Methodology, Data curation, Investigation, Writing—review and editing. WS: Data curation, Investigation, Methodology, Software, Writing—original draft. YY: Formal analysis, Methodology, Writing—review and editing. YW: Data curation, Writing—original draft. ZH: Formal analysis, Writing—original draft. XH: Formal analysis, Methodology, Writing—original draft. RZ: Project administration, Supervision, Writing—review and editing. JY: Conceptualization, Funding acquisition, Visualization, Writing—review and editing. JH: Investigation, Resources, Writing—review and editing.
