# Supplementary material for: Loaded delta-hemolysin shapes the properties of Staphylococcus aureus membrane vesicles
Source: Front Microbiol. 2023 Oct 6;14:1254367. doi: 10.3389/fmicb.2023.1254367 (PMC10588482; doi:10.3389/fmicb.2023.1254367)
Supplement: Supplementary file 1 [file Data_Sheet_1.pdf]

Supplementary Data

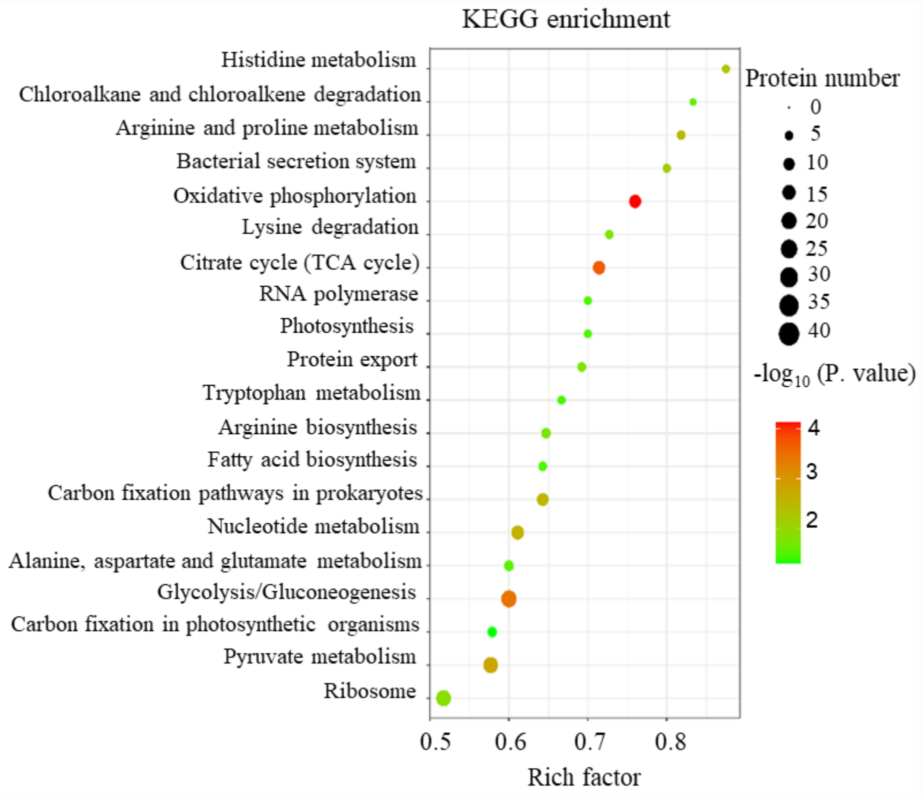

Supplementary Figure 1. Kyoto Encyclopedia of Genes and Genomes (KEGG) pathway analysis of proteins identified in MVs derived from *S. aureus* RN4220 (^{wt}MVs). The horizontal axis shows the Rich factor, which indicates a percentage of the number of differentially expressed proteins annotated to the KEGG pathway among the total number of identified proteins annotated to the same pathway.

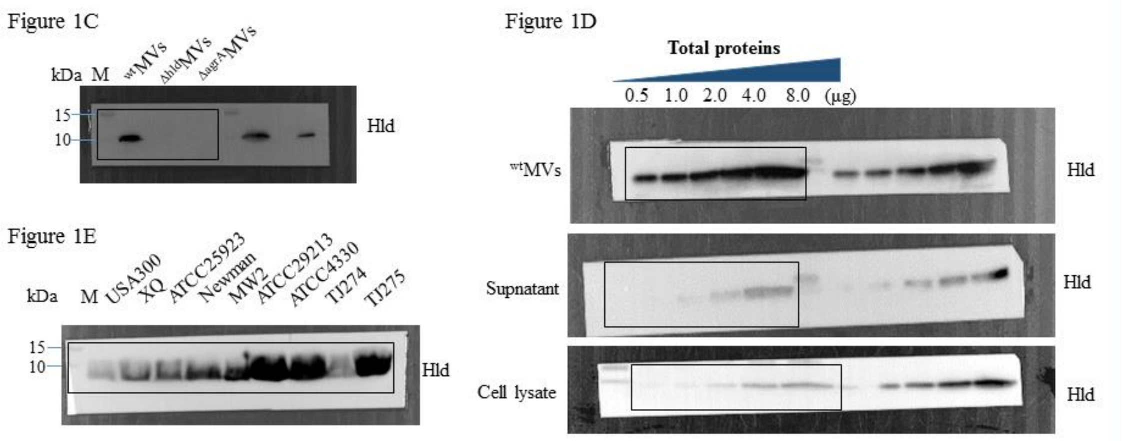

Supplementary Figure 2. Full Western blot data. The full-length blots for the indicated Western blot pictures. The black boxes represent the depicted parts of the blot. Figure 1C is the full length of Fig 1C that showed the identification of enriched Hld in ^{wt}MVs, $^{\Delta hld}$MVs,

and $^{\Delta agrA}$MVs by Western blot. Figure 1D is the full length of Fig 1D that demonstrated Western blot analysis of Hld in ^{wt}MVs, culture supernatant, and cell lysate of RN4220. Figure 1E is the full length of Fig 1E that showed the detection of enriched Hld in the MVs derived from *S. aureus* strains with different genetic lineages with Western blot.

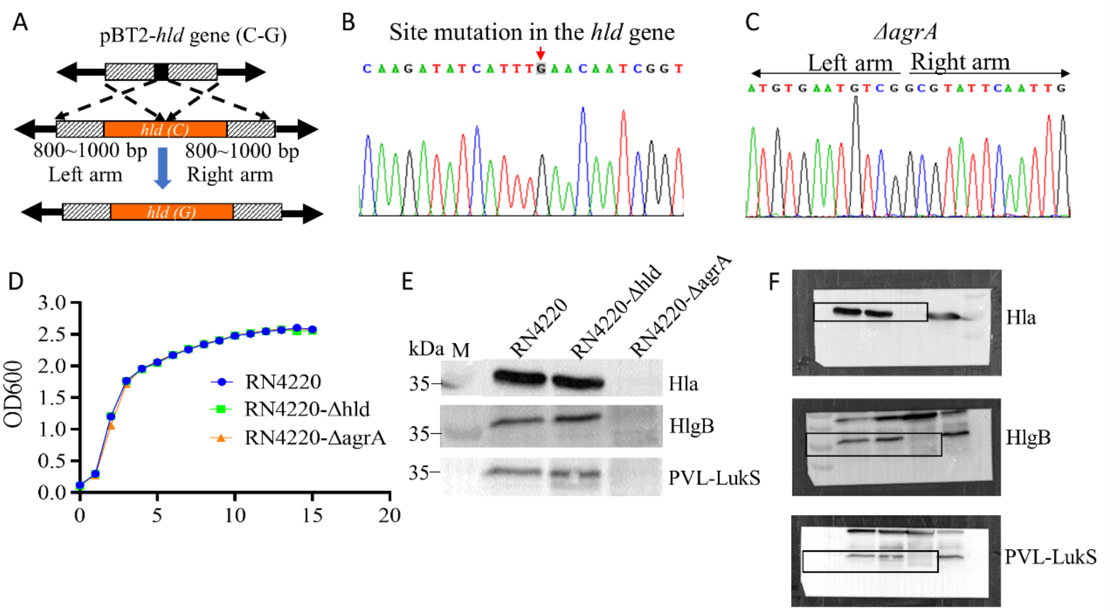

Supplementary Figure 3. Generation and identification of *S. aureus* RN4220-Δhld and RN4220-ΔagrA. (A) The schematic diagram showing the markerless site-mutation of *hld* gene in *S. aureus* RN4220. (B) Identification of RN4220-Δhld. The fragment of the *hld* gene was amplified by PCR, and the product of PCR was sequenced. The change of "C" to "G" was indicated by a red arrow. (C) Sequencing verification of RN4220-ΔagrA mutant. The left and right arms was ligated and the *agrA* gene was deleted. (D) Growth curves of *S. aureus* RN4220 and its derivatives RN4220-Δhld and RN4220-ΔagrA with two independent experiments. (E) Western blot analysis of Agr-regulated virulence factors, such as Hla, HlgB, and PVL-LukS, in ^{wt}MV, $^{\Delta hld}$MVs, and $^{\Delta agrA}$MVs prepared from indicated strains with two independent experiments. The size of proteins was shown by the protein marker (M) on the left. (F) The full-length blots for (E). The black boxes represent the depicted parts of the blot.

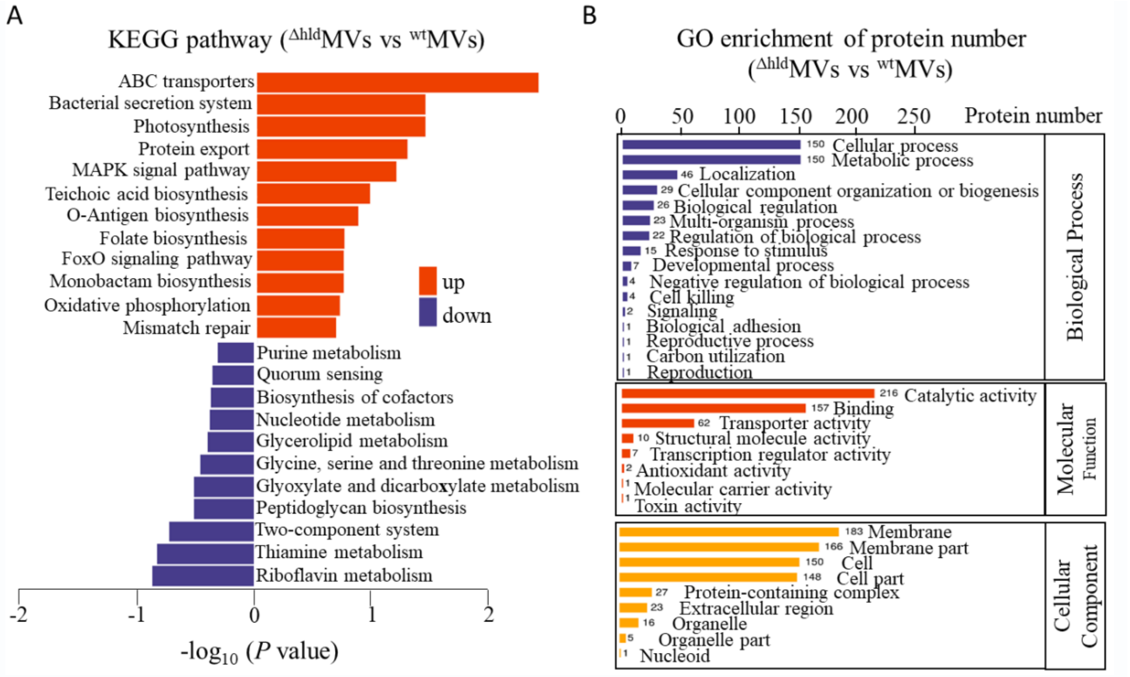

Supplementary Figure 4. Comparative proteomic analysis of ^{wt}MVs and $^{\Delta hld}$MVs. (A) Enrichment butterfly plot of pathways with up- or down-regulated differential proteins in $^{\Delta hld}$MVs vs ^{wt}MVs. (B) GO function analysis of differentially expressed proteins in $^{\Delta hld}$MVs vs ^{wt}MVs.

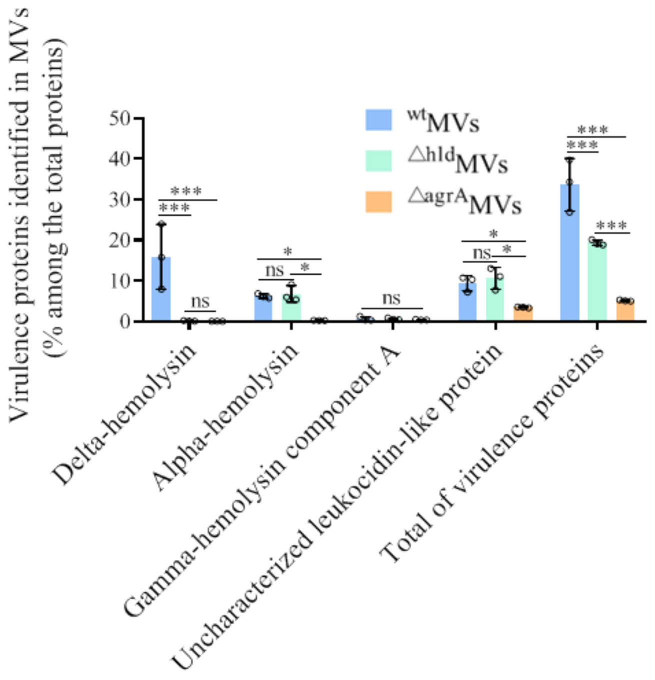

Supplementary Figure 5. Portion of virulence proteins identified in MVs counted by label-free quantity (LFQ) intensity. The data were presented as mean ± SD of three biological replicates. Statistical significance was calculated by One-way ANOVA; ns indicates no statistical significance; **P* < 0.05, ***P*< 0.01, and ****P*< 0.001.

Supplementary Table 1 Strains and plasmids used in this study

Strains and Plasmids	Description	Sources
**Strains**		
S. aureus		
RN4220	NCTC 8325-4, an initial recipient for modification of plasmids prepared from *E. coli*.	(Berscheid et al., 2012)
RN4220-Δhld	RN4220 with site-directed mutation of the *hld* gene to inactivate Hld expression	This study
RN4220-ΔagrA	RN4220 with *agrA* deletion	This study
USA300	ATCC-BAA-1556 (FPR3757), ST8/*agr*-IV, a high-virulent and multidrug-resistant strain extensively used in *S. aureus* animal models	(McDougal et al., 2003)
Newman	NCTC 8178, ST8/*agr*-I, a highly virulent strain that is extensively used in *S. aureus* animal models	(Duthie and Lorenz, 1952)
MW2	HIP 08270, ST1, highly virulent	(Baba et al., 2002)
ATCC25923	Laboratory reference strain, Seattle 1945, *agr*-III	(Yuan et al., 2018)
ATCC29213	Laboratory reference strain	ATCC
ATCC4330	Laboratory reference strain	ATCC
XQ	ST121/*agr*-IV, a clinical isolate, highly virulent	(Liu et al., 2018; Rao et al., 2015)
TJ274	A clinical strain isolated from a patient with coronary heart disease	This study
TJ275	A clinical strain isolated from patient with otitis externa	This study
E. coli		
DH5α	Clone host strain, Amp^r	TransGen
**Plasmids**		
pBT2	Shuttle vector, temp sensitive, Amp^r, Cm^r	(Bruckner, 1997)
pBT2-Δhld	pBT2 recombinant plasmid for site-mutation of the *hld* gene in RN4220	This study
pBT2-ΔagrA	pBT2 recombinant plasmid for knock-out of the *agrA* gene in RN4220	This study
pBT2-tetP/hld	pBT2 recombinant plasmid carrying the wild-type *hld* gene under the control of tetP.	This study
pYT3	Shuttle vector, temp sensitive, Tet^r	(Tamamura et al., 2013)

Amp^r, ampicillin resistant; Cm^r, chloramphenicol resistant; Tet^r, tetracycline resistant.

Supplementary Table 2 Primers used in this study

Primer	Sequence (5'-3')	Descriptions	Reference
**Primers for construction of RN4220-Δhld**			
delta-L	GATATCATTTGAACAATC GGTGAC	Amplification of left arm of the hld gene, the site mutation "G" was introduced in the primer to achieve stop condon "TGA".	This study
pBT2-delta-R (*Bam*HI)	GAGCTCGGTACCCGGGG ATCCAGATAACTCAGTA AGAACCCAT		
pBT2-delta-L (*Sal* I)	CTTGCATGCCTGCAGGT CGACAATAAGTCGCACA GGAATGG	Amplification of right arm of the hld gene.	This study
delta-R	GTCACCGATTGTTCAAAT GATATC		
delta-outer-L	GGCATTCATTACTAAAGG CACG	Verification of *S. aureus* mutant.	This study
pBT2-down	GCTAACGCAGTCAGGCA CC		
pBT2-up	GAGAGTCATTACCCCAG GCGTT	Verification of *S. aureus* mutant.	This study
delta-outer-R	GCTGCGATGTTACCAAT GTTTT		
**Primers for construction of RN4220-tetP/hld and RN4220-Δhld-tetP/hld**			
up-pBT2-eno-tetP-L	TCGAGCTCGGTACCCGG GATGATCGCATTAGACG GTA	Amplification of left arm of the hld gene with tetP promotor.	This study
up-tetP-R	TTGTTGAACGTTATTTAT CTAAGTTATAGAATGATT TGATACCG		
tetP-L	AGATAAATAACGTTCAA CAAACGGGCCAT	Amplification of tetP promotor	
tetP-R	ATGGCACAAGATTTCAC CCTCCAATAATGAGG		
tetP-hld-L	AGGGTGAAATATGGCAC AAGATATCATTTC	Amplification of the hld gene.	This study
tetp-hld-R	GCATTCATTACTAAAGGC ACATAAAGAAAA		
down-eno-L	TAATGAATGCTTTTCTTT ATAATCAAATGCTGAC	Amplification of right arm of hld gene with tetP promotor.	
down-pBT2-eno-R	CTTGCATGCCTGCAGCT GCTTTTACCTTCTTGGAG		This study
eno-outer-L	TGAAATCATCGCACCAG AA	Verification of *S. aureus* mutant (pBT2-down as the reverse primer)	This study
eno-outer-R	TTACATACCAAGATAACT TATGCA	Verification of *S. aureus* mutant (pBT2-up as the forward primer)	This study
**Primers for construction of RN4220-ΔagrA**			

up_agrA (*Bam*H I) fwd	CGCGGATCCCTACAAATACAAGTTCAAAC	Amplification of left arm for the *agrA* deletion.	This study
up_ agrA (Sal I) rev	GATTTACAATTGAATACGCCGACATTCACATCCTTATGGCTAG		
down_ agrA (*Sal* I) fwd	CTAGCCATAAGGATGTGAATGTCGGCGTATTCAATTGTAAATC	Amplification of right arm for the *agrA* deletion.	This study
down_ agrA (*Hind* III) rev	GGGAAGCTTTATGGGATAACGCTGAAGAT		
agrA-outer-L	CATCAGTTTCATTATTAGCGAC	Verification of *S. aureus* mutant (pBT2-down as the reverse primer)	This study
agrA-outer-R	AGTCCGATGAGAGATGCACA	Verification of *S. aureus* mutant (pBT2-up as the forward primer)	This study

Reference

Baba, T., Takeuchi, F., Kuroda, M., Yuzawa, H., Aoki, K., Oguchi, A., et al. (2002). Genome and virulence determinants of high virulence community-acquired MRSA. *Lancet* 359, 1819–1827. doi: 10.1016/s0140-6736(02)08713-5

Berscheid, A., Sass, P., Weber-Lassalle, K., Cheung, A. L., Bierbaum, G. (2012). Revisiting the genomes of the *Staphylococcus aureus* strains NCTC 8325 and RN4220. *Int. J. Med. Microbiol*. 302, 84–87. doi: 10.1016/j.ijmm.2012.01.002

Bruckner, R. (1997). Gene replacement in *Staphylococcus carnosus* and *Staphylococcus xylosus*. *FEMS. Microbiol. Lett*. 151, 1–8. doi: 10.1111/j.1574-6968.1997.tb10387.x

Duthie, E. S., Lorenz, L. L. (1952). Staphylococcal coagulase: mode of action and antigenicity. *J. Gene Microbiol*. 6, 95–107. doi: 10.1099/00221287-6-1-2-95

Liu, H., Shang, W., Hu, Z., Zheng, Y., Yuan, J., Hu, Q., et al. (2018). A novel SigB(Q225P) mutation in *Staphylococcus aureus* retains virulence but promotes biofilm formation. *Emerg. Microbes Infect*. 7, 72. doi: 10.1038/s41426-018-0078-

1

McDougal, L. K., Steward, C. D., Killgore, G. E., Chaitram, J. M., McAllister, S. K., Tenover, F. C. (2003). Pulsed-field gel electrophoresis typing of oxacillin-resistant Staphylococcus aureus isolates from the United States: establishing a national database. *J. Clin. Microbiol.* 41, 5113–5120. doi: 10.1128/JCM.41.11.5113-5120.2003

Rao, Q., Shang, W., Zhou, K., Zhou, R., Rao, X., et al. (2015). Fatal multiple organ failure in an adolescent due to community-acquired methicillin-susceptible *Staphylococcus aureus* ST121/*agr*IV lineage: case report and review. *Revi. Med. Microbiol.* 26, 163–167. doi: 10.1097/MRM.0000000000000050

Tamamura, Y., Tanaka, K., Akiba, M., Kanno, T., Hatama, S., Ishihara, R., et al. (2013). Complete nucleotide sequences of virulence-resistance plasmids carried by emerging multidrug-resistant *Salmonella enterica* serovar typhimurium isolated from cattle in Hokkaido, Japan. *PLoS One* 8, e77644. doi: 10.1371/journal.pone.0077644

Yuan, J., Yang, J., Hu, Z., Yang, Y., Shang, W., Hu, Q., et al. (2018). Safe *staphylococcus aureus* platform for the development of multivalent nanoscale vesicles against viral infections. *Nano Lett.* 18, 725–733. doi: 10.1021/acs.nanolett.7b03893
